# An extract of *Rosaceae,* *Solanaceae* and *Zingiberaceae* increases health span and mobility in *Caenorhabditis elegans*

**DOI:** 10.1186/s40795-022-00498-8

**Published:** 2022-01-13

**Authors:** Samantha Hughes, Nikki Kolsters, David van de Klashorst, Emanuel Kreuter, Karin Berger Büter

**Affiliations:** 1 BioCentre, HAN University of Applied Sciences, 6525EM, Nijmegen, Netherlands; 2 Department of Environment and Health, Vrije Universiteit Amsterdam, De Boelelaan 1085, 1081HV Amsterdam, Netherlands; 3 Bioactive Botanicals Swiss AG, Emeligarten 6, 8592 Uttwil, Switzerland

**Keywords:** *C. elegans*, Health span, Plant extract, Sarcopenia, Muscle, Mitochondria

## Abstract

**Background:**

Members of the *Rosaceae*, *Solanaceae* and *Zingiberaceae* families which include fruits such as cherries, tomatoes and ginger are known to have health promoting effects. There is growing interest in consuming these “functional foods” as a means to increase health and healthy ageing. However, many studies explore the effect of these foods in isolation, not as a blend of multiple functional foods.

**Methods:**

In this study, an extract containing the dried berries, fruits, and roots of members of these families was prepared, which we called Bioact®180. The nematode *Caenorhabditis elegans* was used to evaluate the effects of Bioact®180 on lifespan and health endpoints, including muscle and mitochondria structure and locomotion.

**Results:**

Exposure to the 1000 µg/mL of Bioact®180 extract, containing 4% total phenols, were healthier, as observed by an increase in mean lifespan with and small but significant increase in maximal lifespan. Nematodes exposed to Bioact®180 displayed better mobility in mid-life stages as well as enhanced mitochondrial morphology, which was more comparable to younger animals, suggesting that these worms are protected to some degree from sarcopenia.

**Conclusions:**

Together, our findings reveal that Bioact®180, a blend of fruits and roots from *Rosaceae*, *Solanaceae* and *Zingiberaceae* family members has anti-aging effects. Bioact®180 promotes health and lifespan extension in *C. elegans*, corresponding to functional improvements in mobility.

## Introduction

As humans age, there is a progressive loss of physiological integrity and a decrease in function, together increasing the risk of chronic diseases such as diabetes, cardiovascular disease and cancer [1]. In our ageing society, many more people are paying attention as to how to increase their quality of life and do so via increased consumption of fresh fruits and vegetables. A diet that is rich in foods containing high levels of antioxidants and polyphenols, as found in fresh fruits and berries, has been associated with protection against cardiovascular disease [2, 3] and having anti-microbial [4], anti-inflammatory [5] and anti-cancer [6] properties. More recently, people are turning to dietary supplements as an alternative option for maintaining good health and preventing disease [7]. Studying the health promoting effects of dietary supplements is challenging, but this can be overcome using the 1 mm long nematode *Caenorhabditis elegans*.

*C. elegans* is a non-parasitic genetically tractable model organism that has a simple physiological structure and short life cycle. As nematodes age, they display deterioration of muscle mass and strength (sarcopenia) comparable to an aging human [8]. There is also a reduction in movement that is due to the progressive disorganisation of myofibrils and loss of myosin filaments [8, 9]. Together, these features make *C. elegans* an ideal model to screen natural compounds that extend lifespan and investigate their positive health effects [10–15]. Indeed, there is a whole array of plant bioactive molecules that have been shown to extend the lifespan of *C. elegans* [16] and many of these are already consumed either as the fruit or as a dietary supplement. Strikingly, many of the compounds that have a positive effect on lifespan, also have a positive effect on health span *i.e.,* improving physiological function [14, 17, 18].

Dietary interventions are a non-genetic way to influence health and life span, with many natural bioactive compounds acting via the insulin signalling pathway. The highly conserved insulin signalling pathway is essential to the regulation of lifespan, specifically the transcription factor FoxO [19], which has been shown to be activated and localised to the nucleus where it promotes energy catabolism and stress resistance [20]. In *C. elegans* the sole FoxO homolog, DAF-16, is translocated to the nucleus when nematodes are exposed to functional foods that increase lifespan, including pomegranate [12], purple wheat [21], blueberries [22] and raspberries [15].

To date, the health promoting effects of so-called functional foods have been investigated in isolation. However, there is growing interest in the generation of dietary supplements from blends of different functional foods [23, 24]. To this end, the aim of our study was to investigate the effects of a blend of extracts of fruits, herbs and roots from plants belonging to the *Rosaceae*, *Solanaceae* and *Zingiberaceae* families, which we have termed Bioact®180. The family *Rosaceae* consists of over 3000 species including cherries, plums, strawberries and blackberries that are known to have the best dietary sources of bioactive compounds [25]. Members of this family have exhibited health promoting effects in *C. elegans*, including cherries [10] and *Damnacanthus officinarum* [26]. Similarly, the *Solanaceae* family members also have a positive effect on health when tested in nematodes, specifically tomatoes [27]. Perhaps one of the most famous members of the *Zingiberaceae* family known for health promoting effects is ginger. Ginger, like other herbs and spices, possesses anti-oxidant, anti-inflammatory and anti-carcinogenic properties [17], with extracts of ginger providing lifespan extending effects in *C. elegans* [28].

Here, we have observed the biological effect of Bioact®180 using *C. elegans*, and found that while there was small but significant enhancement to overall lifespan in the nematodes, there was a clear and striking increase in health span. There was no striking activation of the insulin signalling pathway via DAF-16, suggesting the compound acts via an independent or parallel pathway. However, we did observe a positive effect on mobility and maintenance of muscle and mitochondrial structure in *C. elegans* when worms were grown in the presence of the plant extract. Together, our data suggests that Bioact®180 has positive health benefits in the nematode.

## Materials and Methods

### Preparation of Bioact®180 extract

A specially formulated plant extract (Bioact®180) was supplied by Bioactive Botanicals Swiss AG, Uttwil, Switzerland (BBS). It was prepared according to European Pharmacopoeia (Ph. Eur.) standards using 50.42 g dried berries, fruits, and roots of pharmaceutical grade from members of the *Rosaceae*, *Solanaceae* and *Zingiberaceae* families. These were extracted with 40% (m/m) ethanol in a drug to solvent ratio of 9–11:1. The obtained mixture was stirred for 1 h and subsequently stored at 15–18 °C protected from light in an air-tight container. After one week, 50.0 g concentrated fruit juice with a solid content of 51% was added, carefully mixed together and again stored for one week. Then the preparation was filtered over a deep layer cellulose filter (AF 15 Filtrox) to give a dark reddish-brown clear liquid extract. The filtered fluid extract was evaporated to a viscous spissum extract on a rotary evaporator at a bath temperature of 40 °C under reduced pressure and finally dried in a vacuum oven at 40 °C and 25 mbar. The herbal extract is characterized by a drug-extract ratio (DER) of 3–5:1.

### Quantification of total phenolic content

Total phenolic content of Bioact®180 was determined by Folin–ciocalteau reagent using Pyrogallol as a reference, according to the method described in the European Pharmacopoeia for the determination of tannins in herbal drugs. The extract was determined to have a total phenolic content of 4%.

### *Caenorhabditis elegans* strains and maintenance of worms

*C. elegans* strains used in this study were wild type var. Bristol *N2*, *RW1596 (myo-3(st386)*V; stEx30 [*myo-3p*::G*FP* + *rol-6(su1006*)]), *TJ356* [zIs356 IV (p*daf-*16::daf*-*16::gfp; *rol-6*)] and *SD1347* [ccIs4251 I (*myo-3p*::GFP::LacZ::NLS + *myo-3p*::mitochondrial* GFP* + *dpy-20*( +)] provided by the *Caenorhabditis* Genetics Centre (CGC). All strains were maintained at 20 °C on nematode growth medium (NGM) seeded with a lawn of *E. coli OP50*, according to standard protocols [29].

To generate a synchronous worm population, gravid worms were bleached using standard protocols [30]. In brief, gravid worms were subjected to alkaline hypochlorite solution (4 mL 5% sodium hypochlorite, 1 mL 4 M sodium hydroxide, 5 mL dH_2_O) to release the fertilised embryos. Hypochlorite solution was removed by washing with M9 buffer. Eggs were left to hatch overnight at 15 °C in M9 buffer in the absence of a food source, giving rise to a population of synchronised L1 larvae.

### Preparation of Bioact®180 supplemented NGM

The Bioact®180 was provided by BBS as a dry powder. The samples were dissolved in a DMSO/water mixture and then added to the molten NGM to the desired concentration. In all cases, the final DMSO concentration was 0.2%

### DAF-16::GFP localisation

Intracellular localisation of DAF-16 was observed using the strain *TJ356* and based on the method described in Buchter et al. [31]. Synchronised L1 animals were placed on *OP50* seeded plates that had been supplemented with Bioact®180 and allowed to develop to L4. At this point, 100 animals were observed for the distribution of DAF-16::GFP in either the cytoplasm or nucleus, with those nematodes having both cytoplasmic and nuclear localisation of DAF-16::GFP given classified as “intermediate”. This was done using a Leica M165FC microscope (at 460–495 nm excitation and 510-550 nm emission wavelengths) using × 20 magnification. In parallel, 5 L4 worms were transferred to a newly seeded plate supplemented with Bioact®180 and allowed to lay eggs. The next generation (F1) of offspring was allowed to develop to L4 when DAF-16::GFP localisation was observed in 100 animals. In both cases, data is presented as a percentage of the population in each category.

### Lifespan assay

For the analysis of lifespan at 20 °C, synchronised wild type strain, *N2*, was grown to L4 on *OP50* seeded NGM. Synchronised L4 animals were transferred to seeded NGM plates spiked with Bioact®180, which is day 0 of the survival assay. The worms were transferred daily during the reproductive period and every 2 days thereafter. Worms were visually assessed every day for death. If there was no movement, the worm was gently tapped on the head with a platinum wire. The worm was recorded as dead when they failed to respond to this touch. It should be noted that the lifespan experiment could not be performed in a blind fashion due to Bioact®180 causing the NGM to have a slight coloured tint.

Survival curves were generated using GraphPad Prism v9 and analysed with OASIS2 (Online application for survival analysis) [32, 33]. OASIS calculates the percentage mortality, with 100% mortality referring to maximal lifespan and was considered as significant when using the log rank test (Mantel-Cox test) so that *p* < 0.0055 (Bonferroni corrections). The mean lifespan (as a restricted mean, which relates to the area under the survival curve) can be further assessed using the Fisher’s Exact Test where *p* < 0.05 at 50% is significant. A biological repeat of the data was undertaken but not a technical repeat.

### Muscle morphology

To assess the muscle morphology, strain *RW1596* worms were prepared in the same manner as the *N2* animals for lifespan analysis. On days 4 and 8 worms were sacrificed for observation and microscopy (*n* ≥ 85 for each condition). Animals were also maintained for a further week to day 15 post L4 before having their muscle morphology observed, where *n* ≥ 19. In all cases, worms were observed over four independent experiments and the data combined. The morphology of the muscle fibers was classified according to Ryu et al. [18] such that “linear” indicated highly organised muscle fibers which are in parallel lines while the “fragmented” classification was given when there is a high level of disruption and the filaments are non-linear and shortened. Data is presented as a percentage of the population in each category.

### Mitochondrial morphology

Strain *SD1347* was used to observe the mitochondrial network in worms that were exposed to Bioact®180. The experiment was prepared in the same manner as for analysis of lifespan and muscle morphology. On days 4 and 8 worms were sacrificed for observation and microscopy over 2 independent experiments (*n* ≥ 39 for each condition). Mitochondrial morphology was determined as linear, intermediate and fragmented [34, 35]. The mitochondrial were “linear” when the mitochondrial networks were long and connected but when the network was highly disrupted with the mitochondria appearing as swollen short fibers, this was described as “fragmented”. An “intermediate” classification was given when there was a mixture of both long, connected networks with some fragmentation in the same cell. Data is presented as a percentage of the population in each category.

### Body bending assay

Worms were placed in a drop of M9 buffer on an unseeded NGM plate at room temperature. Movies of thrashing worms were recorded using a Leica S8aP0 stereomicroscope with Leica DMC2900 camera and the LAS v4.12 software for 90 s. Bends per minute were obtained with the Worm Tracker plugin (wrMTrck) from the ImageJ software [36]. Graphs were prepared using GraphPad Prism v9. The data was collected in one biological replicate, with *n* ≥ 15 on day 4 and *n* ≥ 10 on day 8.

### Microscopy

Worms are mounted onto 2% agarose pads in 0.1% sodium azide. Fluorescent imaging was carried out using a Zeiss Imager.M2 microscope and photomicrographs were taken using a × 63 objective (Zeiss) and Zeiss Zen 2012 Blue Software. The images of DAF-16::GFP were taken with a Leica DMi8 and LASx software. Representative images of all animals were compiled using Adobe Photoshop 7.0 and Adobe Illustrator.

## Results

### Bioact®180 extends mean and total lifespan of *C. elegans*

There is growing interest in the health promoting benefits of dietary supplements, specifically those which maintain health during mid- to late- life stages [8]. To this end, the aim of our study was to investigate the effects of a preparation comprising the dried berries, fruits, and roots from members of the *Rosaceae*, *Solanaceae* and *Zingiberaceae* families, Bioact®180, on various aspects of health using *C. elegans* as the model organism.

**Fig. 1:**
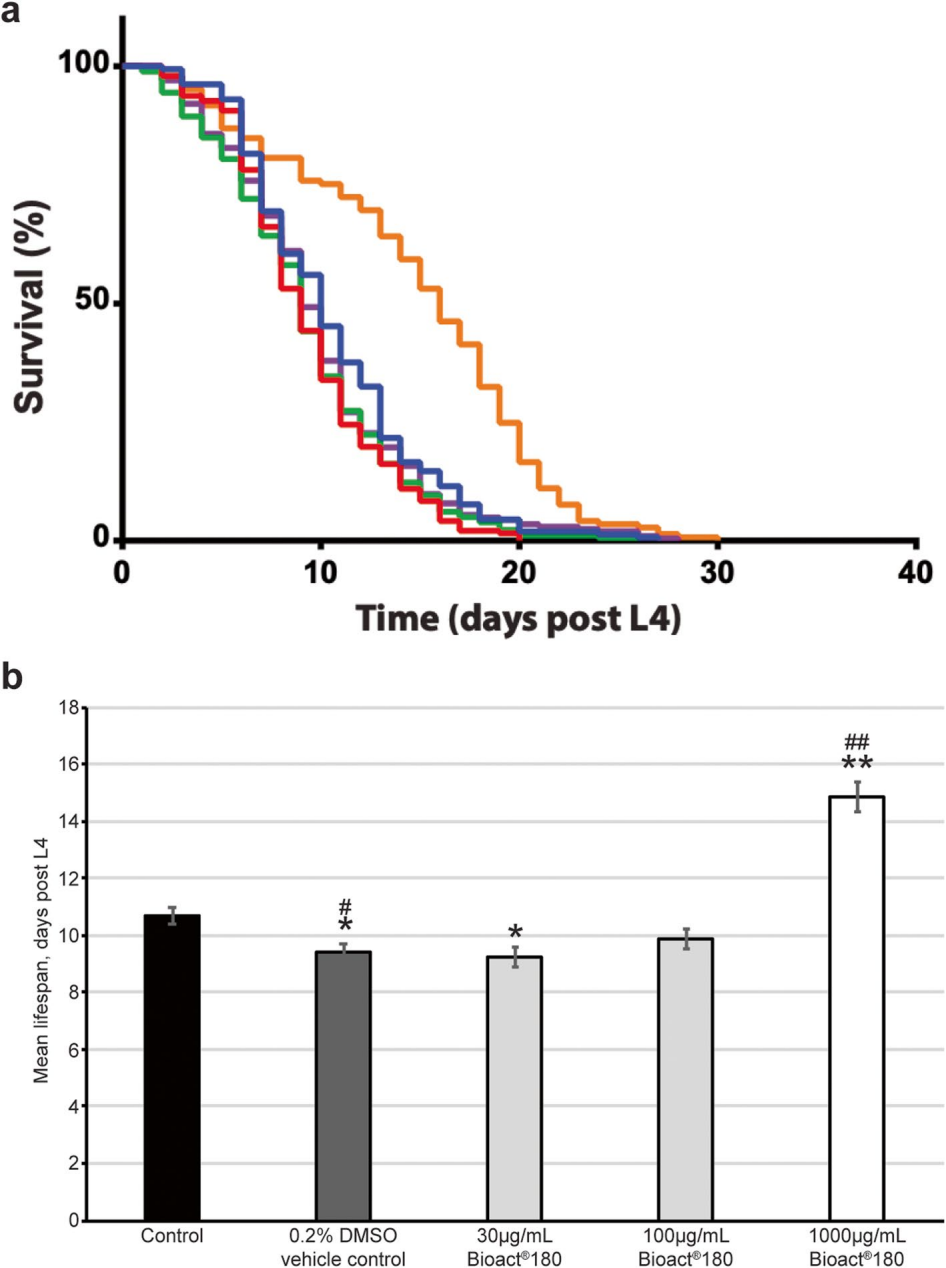
Lifespan is extended when worms are exposed to 1000 µg/mL to Bioact®180. (**a**) Survival curves of wild type (strain, *N2*) animals exposed to Bioact®180 supplemented NGM. The animals in control conditions (blue line; *n* = 276) have a similar curve to those exposed to the vehicle control (0.2% DMSO; red line; *n* = 192), 30 µg/mL Bioact®180 (green line; *n* = 178) and 100 µg/mL Bioact®180 (purple line; *n* = 203). Exposure to 1000 µg/mL Bioact®180 (orange line; *n* = 145) shifts the curve to the right, displaying an increase in health span as well as a slight increase in maximal lifespan. (**b**) The restricted mean lifespan is calculated from the survival curves [32, 33] and plotted here. Error bars represent the standard error of mean (s.e.m.) and statistical differences were compared to the control using the Fisher’s Exact test at 50% mortality, where */^#^*p* < 0.05 and **/^##^*p* < 0.001 and * compares data to the no compound control and # to the DMSO vehicle control. All data is further described in Table 1

We found that wild type worms had a maximal lifespan of 27 days post-L4, while those animals exposed to NGM supplemented with 30 µg/mL Bioact®180 had a slight reduction in maximal lifespan. Conversely, nematodes exposed to elevated concentrations of Bioact®180 (1000 µg/mL) were found to have a small, 11%, but significant increase in maximal lifespan, living for a maximum of 30 days (Fig. 1a and Table 1)**.** The restricted mean lifespan for control animals was 11 days which was significantly increased to 15 days when worms were exposed to 1000 µg/mL Bioact®180 (Fig. 1b, Table 1). This 39% increase in mean lifespan is suggestive that Bioact®180 at elevated doses provides a health promoting benefit. As Bioact®180 had the most significant effect on life span at 1000 µg/mL, we chose to study this dose in more detail.

**Table 1:** Bioact®180 significantly enhances health and lifespan

Bioact®180 concentration	Number of worms	Age in days at 100% mortality	Restricted mean			Bonferroni *p* value	
			**Days**	**Std. Error**	**95% C.I**	**To 0 µg/ml**	**To DMSO**
0 µg/mL	276	27	11	0.28	10.13 ~ 11.23	-	0.0024
DMSO (0.2%)	192	20	9	0.27	8.87 ~ 9.93	0.0024	-
30 µg/mL Bioact®180	178	24	9	0.33	8.58 ~ 9.87	0.0071	1.000
100 µg/mL Bioact®180	203	28	10	0.34	9.18 ~ 10.52	0.4906	0.5754
1000 µg/mL Bioact®180	145	30	15	0.53	13.81 ~ 15.87	0.0000	0.0000

Raw lifespan data was analysed using the OASIS software [32, 33] which provides the restricted mean lifespan (the average survival time obtained from the area under the curve) with standard error and 95% confidence interval (C.I.) and the observed days at which 50% and 100% mortality occurred. The maximal lifespan of control animals was 27 days post L4, and only the highest concentration of Bioact®180 significantly extended this, to 30 days. The restricted mean lifespan was 11 days in control animals compared to 15 days for animals grown in the presence of 1000 µg/mL Bioact®180. The Bonferroni correction was applied to show where the changes to lifespan were most significant. For lifespan plots, see Fig. 1.

### DAF-16::GFP localisation is not disrupted by Bioact®180

**Fig. 2:**
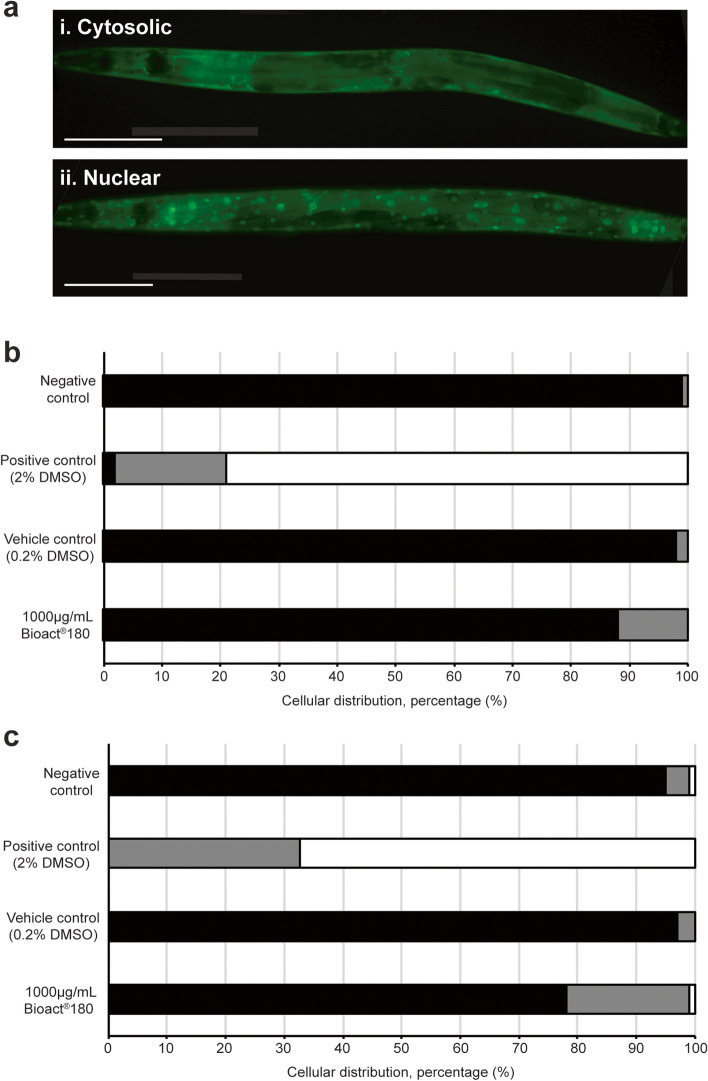
Bioact®180 does not affect the nuclear localisation of DAF-16::GFP. (**a**) Representative images of worms that have (i) cytoplasmic and (ii) nuclear DAF-16::GFP localisation. Scale bars are 100 µm. (**b**) Worms were placed onto Bioact®180 supplemented NGM as L1s and the localisation of DAF-16::GFP observed at the L4 stage. (**c**) From these, 5 random L4 animals were transferred to a fresh Bioact®180 supplemented NGM plate and allowed to lay eggs and then removed. When these progeny were L4, DAF-16::GFP localisation was recorded. Black bars indicate cytoplasmic localisation and white bars show DAF-16::GFP located in the nucleus where it is able to activate the insulin signalling pathway. Grey bars show an intermediate localisation, whereby DAF-16::GFP is found in both the nucleus and cytoplasm. The negative control was NGM, a 0.2% DMSO vehicle control was included together with a 2% DMSO positive control. Strain, *TJ356*; *n* = 100 for all conditions

Lifespan is regulated in humans, rodents, flies and worms by, amongst others, the conserved transcription factor, FoxO [19]. We explored the effect of Bioact®180 on the nuclear translocation of DAF-16, the nematode homolog of FoxO, as a proxy for the positive influence of DAF-16 in lifespan extension. By using a transgenic strain expressing the fusion protein DAF-16::GFP, fluorescence could be detected in the cytoplasm or nucleus (Fig. 2a), with nuclear localisation suggesting activation of the insulin signalling pathway [31]. As expected, under normal conditions, the majority of DAF-16::GFP expression is in the cytoplasm and when C. elegans exposed to 2% DMSO (the positive control), 80% of animals display nuclear localisation of DAF-16::GFP. When *C. elegans* are grown from L1 to L4 in the presence of Bioact®180, no animals have nuclear localisation of the DAF-16::GFP, with the majority having cytoplasmic localisation of DAF-16::GFP and 10% an intermediate localisation pattern i.e. DAF-16::GFP observed in both the nucleus and cytoplasm (Fig. 2b). There is a trend towards a nuclear expression in the next generation of animals (Fig. 2c), with 1% of animals exposed to Bioact®180 having nuclear localisation and 21% of animals displaying an intermediate localisation of the transcription factor.

### Worms are more mobile when exposed to Bioact®180, likely due to better mitochondrial function

**Fig. 3:**
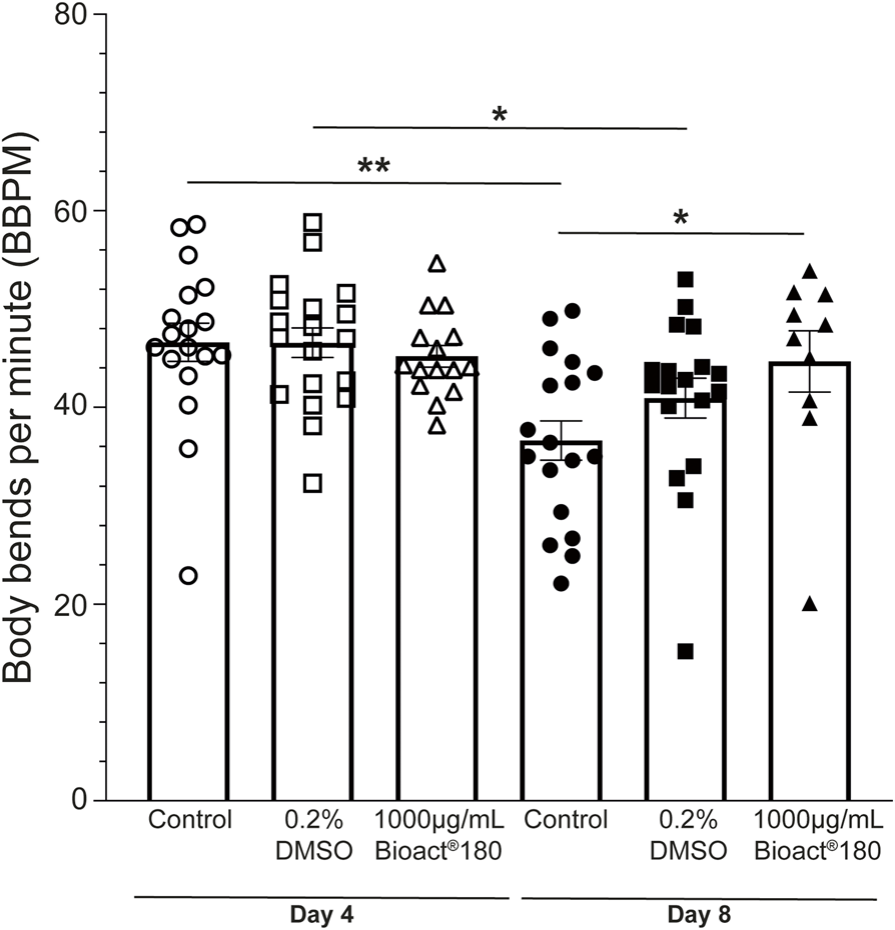
The mobility of animals exposed to Bioact®180 is maintained over time. Worms (strain, *N2*) were placed on no compound control (circles), 0.2% DMSO (squares) or 1000 µg/mL Bioact®180 (triangles) supplemented NGM as L4s (at *T* = 0). The number of head-to-tail body bends were assessed for each condition with the average and standard error of the mean shown. On day 4, worms in control conditions (open circles; *n* = 18) had the same number of body bends per minute, BBPM, as those exposed to DMSO (open squares; *n* = 19) or Bioact®180 (open triangles; *n* = 15). When the worms are 8 days of age, the number of BBPM significantly decreases when worms are exposed to control conditions (closed circles; *n* = 18) and the DMSO vehicle control (closed squares; *n* = 18). In contrast, worms on 1000 µg/mL Bioact®180 (closed tringles; *n* = 10) show no change in BBPM, suggesting that the compound is providing some health benefit. *p*-value obtained by the 2-tailed 2-sample t-test, where *p < 0.05 and **p < 0.001. If no asterisks, there is no significant difference

Health span is the period in adulthood without any physical impairment that precedes senescent decline [8] and is the period where an individual is in good health and free of disease [37]. Mobility is a powerful indicator of health, with worms moving less as they age, similar to humans [38]. As the mean and maximal life span in worms exposed to 1000 µg/mL Bioact®180, was increased, we wished to explore the positive effect on health by exposure to Bioact180 with the observation of mobility. To do this, the number of head-to-tail body bends (thrashing) was assessed in wild type worms (strain, *N2*) on day 4 and 8 post-L4. There was no significant difference in the number of body bends on day 4 between control and Bioact®180 exposed animals, with worms at all conditions having 46 body bends per minute, BBPM (Fig. 3). In contrast, significant differences were observed on day 8. The no compound control displayed a 37 BBPM on day 8, a significant reduction (*p* = 0.001) compared to worms at day 4. Interestingly, the DMSO control had a similar thrash rate to control worms on day 4 (47 BBPM) which was significantly reduced to 41 BBPM (*p* = 0.03) on day 8, suggesting DMSO alone has the ability to improve mobility. Strikingly, animals exposed to 1000 µg/mL Bioact®180 displayed no impairment of thrashing, with worms displaying 45 BBPM on day 4 and on day 8 (*p* = 0.854). This further confirms that the Bioact®180 is causing nematodes to have an enhanced health span.

**Fig. 4:**
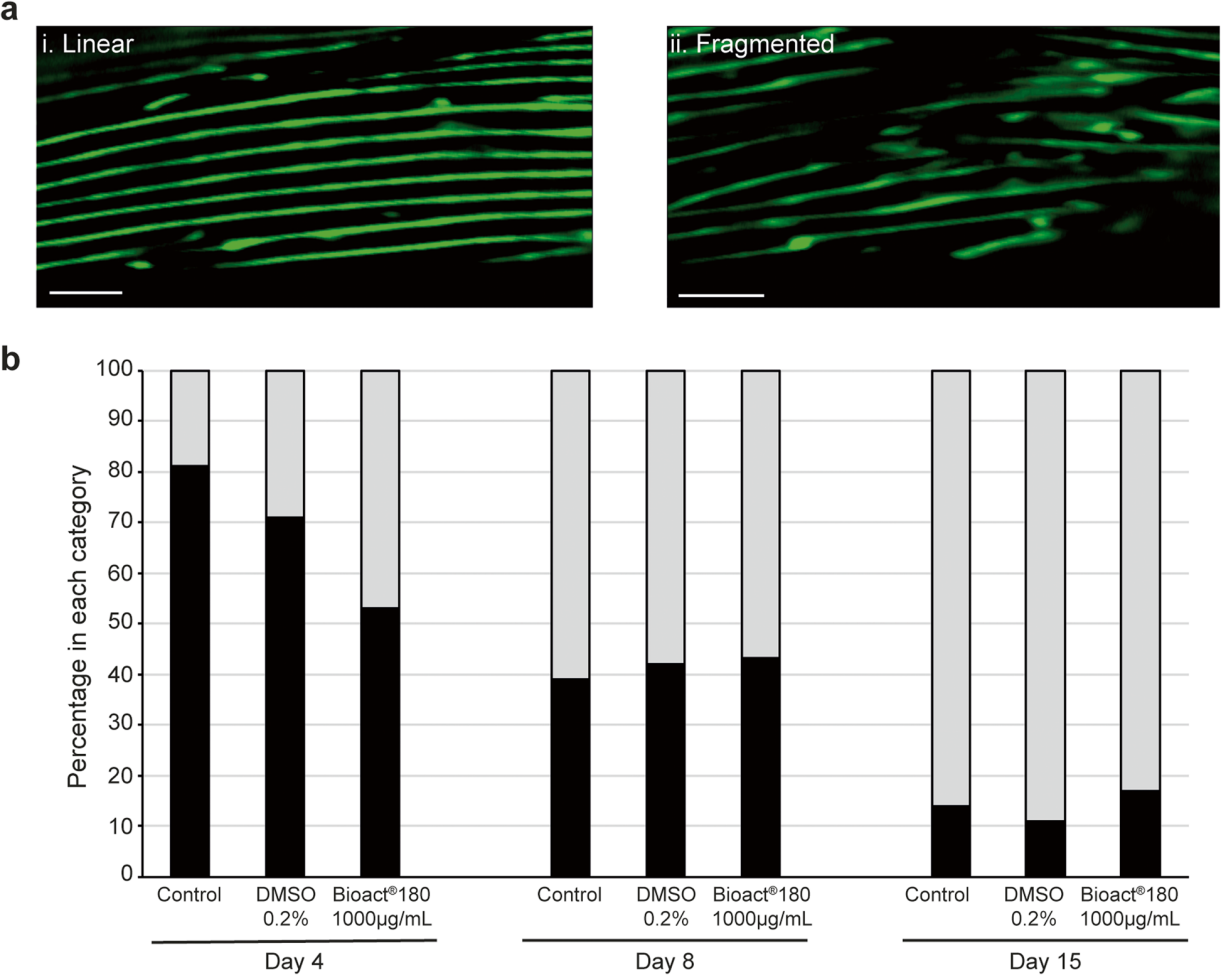
Muscle morphology is unchanged in Bioact®180 exposed worms compared to wild type. Worms (strain, *RW1596*) were placed on no compound control, 0.2% DMSO or 1000 µg/mL Bioact®180 supplemented NGM as L4s (at T = 0). The muscle morphology was observed via the *myo-3*p::GFP reporter for each condition on days 4, 8 and 15 and assessed as being linear or fragmented. (**a**) Representative image of a worm displaying (i) linear or (ii) fragmented muscle fibres. Scale bars, 100 µm. (**b**) Graph to show the percentage of worms in each category at the different time points. Black bars indicate linear muscle morphology while the grey bars are for those fibres which are fragmented. Data was from 4 independent biological replicates with more than 85 worms assessed on day 4 (control *n* = 88; DMSO *n* = 85; Bioact®180 *n* = 85) and more than 95 worms on day 8 (control *n* = 110; DMSO *n* = 95; Bioact®180 *n* = 93), and around 20 worms on day 15 (control *n* = 28; DMSO *n* = 19; Bioact®180 *n* = 24)

Next, we visualised the sarcomere structure of the Bioact®180 exposed animals to determine if the differences in mobility could be attributed to changes in muscle structure. As worms age, sarcopenia occurs, where the muscle fibre organisation deteriorates becoming less parallel and more fragmented (Fig. 4a) [8, 9]. Using a strain expressing a *myo-*3p::GFP fluorescent reporter (*RW1596*), we observed the appearance of the muscle fibres on day 4, 8 and 15 of exposure to Bioact®180. Under control conditions (no compound and 0.2% DMSO vehicle control), the majority of the muscle fibres had a regular, smooth appearance, which become more fragmented as the worms age (Fig. 4b). In contrast, the worms exposed to 1000 µg/mL Bioact®180, had similar numbers of worms with regular and irregular muscle fibres on days 4 and 8 while by day 15 there was no difference in muscle morphology between exposed and non-exposed worms. Overall, our data shows that the elevated thrash rate of worms on the extract is likely to be result of a different mechanism(s) than enhanced muscle fibre, such as enhanced mitochondrial function.

**Fig. 5:**
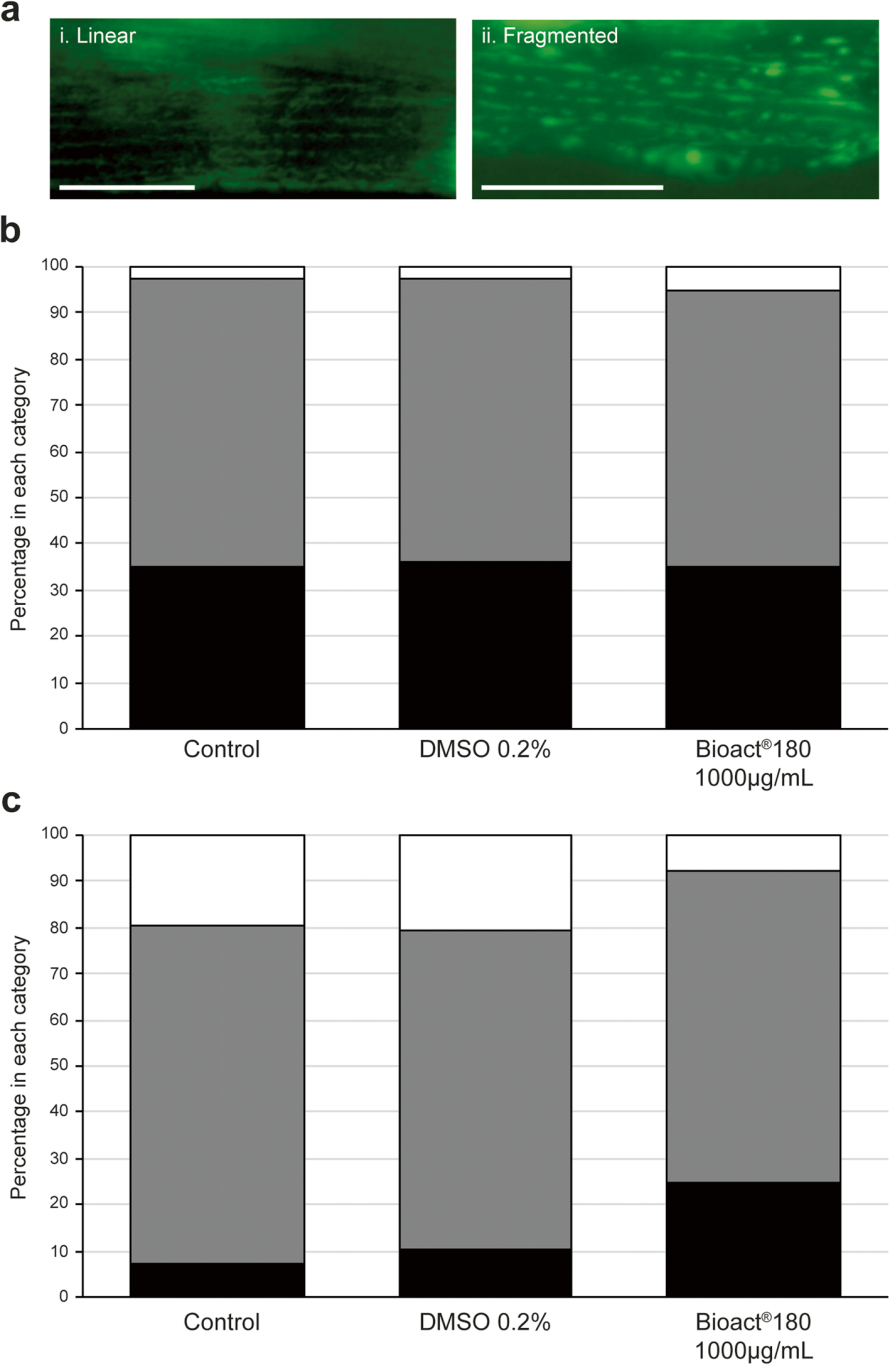
Mitochondrial morphology of worms exposed to Bioact®180 have less degradation on day 8 of life. Worms (strain, *SD1347*) were placed on no compound control, 0.2% DMSO or 1000 µg/mL Bioact®180 supplemented NGM as L4s (at T = 0). The mitochondrial network of worms was assessed for linear, intermediate or fragmented morphology on day 4 and 8. (**a**) Representative image of a worm displaying a (i) linear or (ii) fragmented mitochondrial network. Scale bars, 100 µm. Graphs to show the mitochondrial morphology on (**b**) day 4 and (**c**) day 8. The classifications are as follows: linear (black bars) where the mitochondrial network has even linear lines; fragmented (white bars) is where the network has reduced significantly and is mostly broken; an intermediate classification (grey bars) is where the network is uneven and starting to fragment. The mitochondrial morphology was assessed over 2 independent experiments, with a total of *n* = 40 for day 4 control, day 4 Bioact®180 and day 8 Bioact®180; *n* = 38 for the DMSO control at day 4 and 8; *n* = 41 for control worms on day 8

The morphology of the mitochondria was observed to gain an insight into their function. During aging the mitochondrial network fragments as a consequence of the aging process [35]. When exposed to conditions that increase lifespan, the aging process slows and hence mitochondrial fragmentation occurs more slowly [35]. Worms expressing a mitochondrial targeted GFP in the body-wall muscle cells (Strain, *SD1347*) were exposed to 1000 µg/mL Bioact®180 from L4 stage and the mitochondrial morphology observed on days 4 and 8. Morphology of the mitochondria were defined according to Regmi et al*.* [35] and Momma et al. [34], with slight modifications: long linear networks were classified as linear and a fragmented classification was given to those networks where the mitochondria were short, more round and highly disconnected from the network (Fig. 5a). An intermediate classification was given to those animals which were neither fully linear nor fully fragmented.

At L4 stage, worms display a linear organisation of the mitochondrial network. As the worms age, the network starts to fragment, with just 35 % of control animals having linear mitochondria at day 4, with DMSO vehicle control and 1000 µg/mL Bioact®180 exposed worms displaying similar morphologies (Fig. 5b). A small number of animals (2–5 %) displayed a fragmented mitochondrial network on day 4. We also observed worms at day 8, where there were clear differences in worms exposed to Bioact®180 (Fig. 5c). Both the negative control and DMSO control conditions had the majority of number of animals displaying defective mitochondrial network, with 20 % of worms having a fragmented network. In contrast, Bioact®180 caused just 8 % of worms to display a fragmented mitochondrial network, with 25 % of worms maintaining linear mitochondrial morphology, suggesting these worms are more able to function normally.

## Discussion

There is growing interest in the health promoting benefits of dietary supplements, specifically those which maintain health during mid- to late- life stages [8]. Bioact®180 is a blend of extracts from the fruits, herbs and roots of plants belonging to the *Rosaceae*, *Solanaceae* and *Zingiberaceae* families and is a rich source of polyphenols. The aim of our study was to investigate the effects of Bioact®180 on various aspects of health using *C. elegans* as the model organism. *C. elegans* is one of the principal models to study ageing due to its short lifespan of 3 weeks and as the nematode age in a similar manner to humans, they can be used to define interventions that promote a long and healthy life.

Bioact®180 had a small but significant effect on maximal lifespan, with worms exposed to 1000 µg/ml Bioact®180 having a large increase in mean lifespan, which was increased from 11 to 16 days compared to non-exposed nematodes. Our experiment, similar to the vast majority of lifespan assays, were not performed in a blinded or randomised fashion, which could limit the objectivity of the results [39]. However, as we performed the assay on a large number of worms, and the statistics are sound, we are confident in the lifespan benefit provided by Bioact®180.

Typically, the insulin-like signalling pathway modulates aging and longevity, which has DAF-16 as a central regulator. We explored the localisation of DAF-16 following exposure to Bioact®180 as a proxy for the positive influence of this transcription factor in lifespan extension. In our experiments, we performed the exposure from L1 to L4 in line with other studies [31, 40] and again in the progeny of these parents. In both cases, we observed no significant shift of DAF-16::GFP to the nucleus upon exposure to Bioact®180. It should be noted that the timing of exposure to compounds is of importance as is the age of measurement of the outcome [39]. Therefore, we cannot exclude that DAF-16 localisation is altered in older animals. An interesting future experiment would be to explore the localisation of DAF-16 in worms throughout their lifespan, but particularly at the point of 50 % mortality, where there are clear differences between the control and Bioact®180 exposed nematodes. However, in *C. elegans* the insulin signalling pathway receptor DAF-2 directs the phosphorylation of DAF-16 via the insulin-activated kinase Akt (AKT-1 and AKT-2) to prevent DAF-16 accumulation in the nucleus [41]. Therefore, it is possible that while we do not observe the nuclear localisation of DAF-16, this is due to the activity of AKT-1 and AKT-2. Further, phosphorylation of DAF-16 is also possible by AMPK independent of the sub-cellular localisation of DAF-16 [42]. Dissecting which of these pathways might be activated by the presence of Bioact®180 is beyond the scope of this work, but as the individual components of Bioact®180 have previously been shown to activate AMPK [43–45], we postulate that this is the case here. AMPK is able to coordinate mitochondrial homeostasis [46] and this can induce autophagy, mitochondrial biogenesis and expression of anti-oxidant enzymes, which are able to improve cellular function [46] and many phytochemicals have been shown to act in this way [16, 47]. We suspect that the mode of action of Bioact®180 is via mitophagy, a specific form of autophagy whereby the cells eliminate damaged mitochondria [48, 49].

There is no comprehensive definition of health span [50] but it is generally defined as the period in adulthood without any physical impairment that precedes senescent decline [8, 37, 51]. We found that the mean lifespan was extended in worms exposed to Bioact®180 and was accompanied by the enhanced mobility of worms. While DMSO alone appeared to improve mobility, this is likely to be a consequence of changes in the permeability of the membrane [52]. The fact that exposure to Bioact®180 prevents a decline in mobility, suggests that the compound has positive effects beyond the ability of DMSO. The organisation of the musculature did not display a more youthful appearance, suggesting that Bioact®180 exerts its effects via other mechanism(s), for example mitochondrial autophagy or inhibition of protein degradation. Mitochondrial morphology in nematodes that had been exposed to Bioact®180 throughout life was observed. We found that the fragmentation processes was occurring more slowly compared to control conditions, further supporting the hypothesis that Bioact®180 has a role in the mitophagy process, whereby cells eliminate damaged mitochondria [48, 49]. It is therefore plausible that Bioact®180 induces mitophagy. A similar compound, Urolithin A, found in members of the *Rosaceae* family (*e.g.*, strawberries, raspberries and blackberries) was also found to result in increased mobility and result in an improvement to muscle cell quality [18], which we also observe after 8 days of exposure to Bioact®180 compound.

## Conclusion

Members of the *Rosaceae, Solanaceae* and *Zingiberaceae* families contain different compound classes that are known to have a positive effect on health. For example, *Solanaceae* are a source of carotenoids, *Rosaceae* is a source for ellagitannin and anthocyanins and *Zingiberaceae* provides phenylpropanoid-derived compounds. Our aim was to generate a mixture, Bioact®180, which had all the benefits of these compounds. In summary, we have shown that Bioact®180 promotes health and lifespan extension in *Caenorhabditis elegans*, corresponding to functional improvements in mobility. However, the exact mechanism(s) by which Bioact®180 functions remain(s) elusive. It is highly likely to be via AMPK activation and/or mitophagy however further work is needed to explore these complex mechanisms, which is currently beyond the scope of this investigation.

## Availability of data and materials: 

The data generated or analysed during this study are included in this article. Raw data could be provided upon request to the corresponding author.
